# Lipid Accumulation Product as a Predictor of Prediabetes and Diabetes: Insights From NHANES Data (1999–2018)

**DOI:** 10.1155/2024/2874122

**Published:** 2024-11-11

**Authors:** Yan Wang, Xiaolan Wang, Ling Zeng

**Affiliations:** ^1^Department of Critical Care Medicine, West China Hospital, Sichuan University, Chengdu, China; ^2^West China School of Nursing, Sichuan University, Chengdu, China

**Keywords:** diabetes, lipid accumulation product (LAP), logistic regression, NHANES, prediabetes

## Abstract

**Background:** The study investigates the association between lipid accumulation product (LAP) and the risk of prediabetes and diabetes. LAP, a measure indicating lipid overaccumulation, is hypothesized to be a significant predictor for these conditions. This research utilizes data from the National Health and Nutrition Examination Survey (NHANES) conducted between 1999 and 2018.

**Methods:** The study followed a structured methodology, starting with data extraction from the NHANES database. Participants' eligibility was determined based on specific inclusion and exclusion criteria, resulting in a final sample size of 24,121 individuals. LAP was calculated using established formulas for men and women. The diagnosis of prediabetes and diabetes was based on standard medical criteria, including HbA1c levels, fasting plasma glucose, and oral glucose tolerance test (OGTT) results. Covariates like demographic variables, lifestyle factors, and other health indicators were also considered. Statistical analysis involved categorizing LAP into quartiles and employing logistic regression models to examine the relationship between LAP and the risk of prediabetes and diabetes.

**Results:** Participants in the highest LAP quartile exhibited distinct characteristics: older age, lower education levels, more former smokers and drinkers, higher blood pressure and cholesterol levels, and greater use of medications. A positive association was observed between LAP and the incidence of prediabetes and diabetes across all models. Specifically, each 10-unit increase in LAP was linked to a 22% increase in risk. Nonlinear relationships were also explored, revealing an inflection point in the risk correlation at an LAP value of 68.1.

**Conclusion:** The study concludes that LAP is a significant predictor of prediabetes and diabetes risk, with higher LAP levels correlating with increased risk. This finding underscores the potential of LAP as a useful marker in identifying individuals at higher risk for these conditions. It also highlights the importance of considering LAP in preventive health strategies.

## 1. Introduction

Diabetes and prediabetes are major global health concerns, largely due to lifestyle changes and an aging population. According to World Health Organization criteria, the prevalence of diabetes in Chinese adults is 11.2%, whereas the American Diabetes Association criteria indicate detection rates of diabetes and prediabetes in Chinese adults at 12.8% and 35.2%, respectively [1]. In the United States, the unadjusted estimated prevalence of diabetes is 14.6%. Alarmingly, 23.3% of these adult diabetes cases were not diagnosed in a timely manner [2]. The International Diabetes Federation's (IDF) Diabetes Atlas reveals that approximately 6.7 million people were expected to die from diabetes or its complications in 2021 [3]. This underscores the importance of early detection of prediabetes and diabetes for effective management and prevention of further disease progression.

Numerous studies have evaluated various biomarkers and their associations with prediabetes and diabetes. Traditional metrics such as the waist-to-height ratio, body mass index (BMI), and waist circumference (WC) have been widely studied for their predictive capabilities. However, these measures often exhibit limitations, including varying accuracy across different populations and age groups. In contrast, the lipid accumulation product (LAP), which combines WC and fasting triglyceride (TG) levels, offers a potentially more robust assessment of metabolic health. Initially proposed by Kahn as a biomarker to identify cardiovascular risk [4], subsequent studies have increasingly supported LAP as an important indicator of metabolic risk [5–7].

Research has also highlighted the role of LAP in predicting diabetes. For example, Liu et al. found that each unit increase in LAP corresponds to a 76.8% increase in diabetes risk [8]. Similarly, Lin et al. reported a significant increase in diabetes risk associated with higher LAP levels in Chinese youth [9]. Cybulska et al. demonstrated the utility of LAP in predicting diabetes and prediabetes in postmenopausal women [10], while Ahn et al. confirmed the reliability of LAP as a screening parameter for prediabetes and diabetes in a German population [11]. Despite these findings, many studies have assumed a linear relationship between LAP and diabetes or prediabetes. However, evidence suggests that the relationship between similar biomarkers and these conditions may be nonlinear [12, 13]. Given the higher prevalence of prediabetes compared to diabetes [14], this discrepancy warrants further investigation.

Our study utilized data from the National Health and Nutrition Examination Survey (NHANES) covering the years 1999–2018. The NHANES, a detailed program assessing the health and nutritional status of the US population, offers an extensive database ideal for research. Our research focused on investigating potential nonlinear relationships between LAP and diabetes and prediabetes. We considered a range of variables, including demographic factors, laboratory test results, and other health-related aspects. By addressing literature gaps, our study is aimed at clarifying LAP's potential as an indicator of prediabetes and diabetes, exploring their complex relationships. This research contributes to the development of more effective diabetes prevention strategies.

## 2. Methods

### 2.1. Study Design and Participants

In our study, we leveraged data from the NHANES, a valuable database spanning from 1999 to 2018. NHANES, a key project of the Centers for Disease Control and Prevention, is aimed at evaluating the health and nutritional status of the US population. It does this through a unique blend of personal interviews and detailed physical examinations, collecting comprehensive health-related data from a diverse cross section of adults and children in the United States.

**Figure 1:**
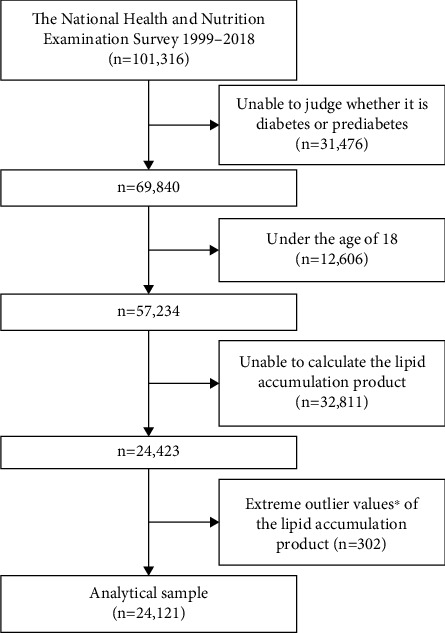
Flowchart of the study. ⁣^∗^Extreme outlier values, defined as those over 3 standard deviations from the mean.

From this extensive database, our initial dataset included 101,316 samples gathered over these years. To refine our analysis, we excluded certain subsets from this pool: 31,476 individuals who were not clearly classified as having diabetes or prediabetes, 12,606 individuals below 18 years of age, 32,811 individuals who lacked calculable LAP values, and 302 individuals as extreme outliers. Consequently, our final study sample was composed of 24,121 participants, carefully selected to ensure a focused and relevant examination of the data (Figure 1).

### 2.2. Calculation of LAP

The LAP was calculated differently for men and women. For men, LAP was calculated as (WC in centimeter − 65) × TG concentration in millimoles per liter. For women, the formula was (WC in centimeter − 58) × TG in millimoles per liter [4].

### 2.3. Assessment of the Diagnosis of Prediabetes and Diabetes

Prediabetes was determined using one or more of these criteria: a healthcare professional's diagnosis, a hemoglobin A1c (HbA1c) level of 5.7% to less than 6.5%, a fasting plasma glucose (FPG) level of 5.6–7.0 mmol/L, or a 2-h FPG of 7.8–11.0 mmol/L during an oral glucose tolerance test (OGTT).

Diabetes was identified if a participant had any of the following: a confirmed medical diagnosis, an HbA1c level over 6.5%, an FPG level of 7.0 mmol/L or more, a random blood glucose level above 11.1 mmol/L, or a 2-h OGTT result exceeding 11.1 mmol/L.

### 2.4. Covariates

In this study, we evaluated demographic variables, lifestyle factors, anthropometric measurements, and laboratory tests through computer-assisted personal interviews. Demographic data included age, gender, race/ethnicity, education level, poverty income ratio (PIR), and medication history. Lifestyle factors covered smoking, drinking, and physical activity. We measured blood pressure during physical exams, and laboratory tests included TG, total cholesterol (TC), and estimated glomerular filtration rate (eGFR). Smoking status was categorized into never smokers (under 100 cigarettes lifetime), former smokers (over 100 cigarettes but stopped by survey time), and current smokers (over 100 cigarettes and still smoking) [15]. Current drinking was classified into heavy (≥ 3 drinks/day for women, ≥ 4 for men, or ≥ 5 binge drinking days/month), moderate (≥ 2 drinks/day for women, ≥ 3 for men, or twice monthly binge drinking), and mild (all other cases) [16]. PIR was defined as low (below 1.3), medium (1.3 to 3.5), and high (above 3.5) [17]. Physical activity was assessed by multiplying weekly activity minutes by their metabolic equivalents (METs) from the Compendium of Physical Activities, yielding MET/week. Activity levels were categorized into low (< 600 METs/week), moderate (600–1199 METs/week), and vigorous (≥ 1200 METs/week) [18]. eGFR was calculated using the 2009 serum creatinine (SCr)–based Chronic Kidney Disease Epidemiology Collaboration—eGFR equation [19].

The detailed methods for calculating covariates such as smoking status, alcohol consumption, and physical activity, as well as other relevant variables, are provided in supporting information (see Tables S1–S4 for details).

### 2.5. Statistical Analysis

The analysis categorized LAP into four quartiles for study: Q1 (0.21–22.34), Q2 (22.34–41.51), Q3 (41.51–72.63), and Q4 (72.63–251.02). Continuous variables conforming to a normal distribution were described using the mean and standard deviation. In contrast, variables with a skewed distribution were represented using the median and interquartile range. Categorical variables were presented as percentages. The study employed one-way ANOVA or Kruskal–Wallis tests for comparing continuous variables, while chi-square tests were used for categorical variables.

The research explored the association between LAP and the risk of prediabetes and diabetes in the general population using multivariable logistic regression analysis. This included three models: Model 1 (unadjusted), Model 2 (adjusted for age, sex, race/ethnicity, and education), and Model 3 (which included all variables in Model 2, plus smoking, alcohol consumption, PIR, METs/week, systolic blood pressure (SBP), eGFR, TG, TC, and the use of lipid-lowering and antihypertensive medications).

To ensure the reliability of conclusions, various sensitivity analyses were conducted. LAP was treated as a categorical variable based on quartiles, and a *p* for trend was calculated to evaluate the results for LAP as a continuous variable and to check for nonlinearity. A generalized additive model (GAM) with smooth curves was used to validate the results, considering continuous variables as curves in the equation. A two-piece logistic regression model was constructed to accommodate nonlinear relationships, analyzing data on either side of the inflection point. The most suitable model for the LAP–diabetes/prediabetes risk relationship was determined using the log-likelihood ratio test.

Additionally, interaction terms were added to the regression models to assess potential interactions between LAP, prediabetes, and diabetes, followed by stratified analysis. Statistical analyses were performed using R (Version 4.2.0) and EmpowerStats, considering a *p* value below 0.05 as statistically significant.

## 3. Results

### 3.1. Baseline Characteristics

Table 1 presents the baseline characteristics of the study participants, divided according to their LAP levels. Notably, individuals in the top LAP quartile (Q4) were generally older, predominantly non-Mexican White, had lower educational levels, and were more likely to be former smokers and drinkers. This group also faced more economic challenges, as reflected in their lower PIRs. From a health perspective, they exhibited higher SBP and diastolic blood pressure (DBP) and increased TC and TG levels, along with a decreased eGFR. Additionally, there was a greater use of antihypertensive and lipid-lowering medications in this cohort. Importantly, a significantly higher incidence of diabetes and prediabetes was found in individuals with higher LAP (*p* < 0.05 for both conditions).

### 3.2. Association Between LAP and Prediabetes and Diabetes

Our analysis demonstrated a consistent positive correlation between LAP levels and the incidence of prediabetes and diabetes across all models of adjusted multivariate logistic regression. In Model 1, the odds ratio (OR) was 1.16 with a 95% confidence interval (CI) of 1.15–1.16; in Model 2, the OR was 1.13 with a 95% CI of 1.12–1.14; and in Model 3, the OR was 1.22 with a 95% CI of 1.20–1.25. Notably, in the fully adjusted Model 3, every 10-unit increase in LAP was associated with a 22% higher risk of these conditions. This trend persisted when LAP was categorized into quartiles. Compared to the lowest quartile (Q1), the odds of developing prediabetes and diabetes were significantly higher in the higher quartiles (Q2: OR = 1.64, 95% CI: 1.46–1.83; Q3: OR = 2.69, 95% CI: 2.38–3.04; and Q4: OR = 5.14, 95% CI: 4.34–6.07), showing a linear increase in risk. This was further supported by a *p* for trend < 0.001 (Table 2).

**Figure 2:**
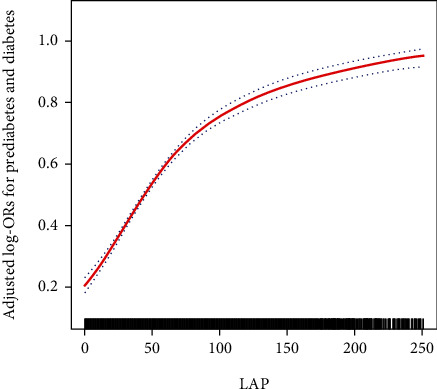
The association between LAP and the risk of prediabetes and diabetes. Age, sex, race/ethnicity, education level, smoking, drinking, poverty income ratio, METs/week, SBP, eGFR, TG, TC, lipid-lowering medications, and antihypertensive medications were adjusted. Abbreviations: LAP, lipid accumulation product; MET, metabolic equivalent of task; SBP, systolic blood pressure; eGFR, estimated glomerular filtration rate; TG, triglyceride; TC, total cholesterol.

Additionally, using GAMs and curve fitting, we explored the potential nonlinearity in the relationship between LAP and the risk of prediabetes and diabetes. Particularly in the fully adjusted model, a nonlinear relationship was evident (Figure 2). The two-piece logistic regression analysis identified an inflection point at an LAP value of 68.1. Below this point, the adjusted OR was 1.32 (95% CI: 1.29–1.35), while above it, the OR decreased to 1.14 (95% CI: 1.11–1.16). The log-likelihood ratio tests confirmed a statistically significant difference in the relationship's slopes on either side of this point (*p* < 0.001), as detailed in Table 3.

**Figure 3:**
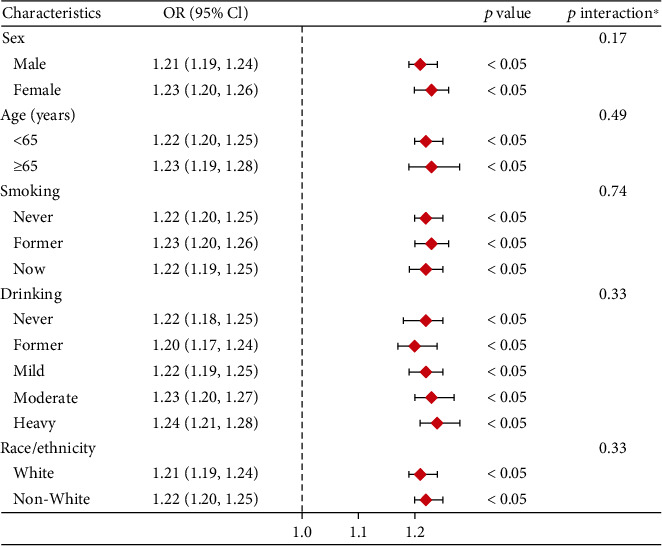
Stratified analyses between LAP/10 and the prevalence of prediabetes and diabetes. ⁣^∗^Each stratification adjusted for all the factors (age, sex, race/ethnicity, education level, smoking, drinking, poverty income ratio, METs/week, SBP, eGFR, TG, TC, lipid-lowering medications, and antihypertensive medications) except the stratification factor itself. Abbreviations: OR, odds ratio; CI, confidence interval; LAP, lipid accumulation product; MET, metabolic equivalent of task; SBP, systolic blood pressure; eGFR, estimated glomerular filtration rate; TG, triglyceride; TC, total cholesterol.

The forest plots, as depicted in Figure 3, indicated consistent interactions across all stratified variables. These plots showed positive associations between LAP levels and the incidence of prediabetes and diabetes in all stratified analyses.

**Figure 4:**
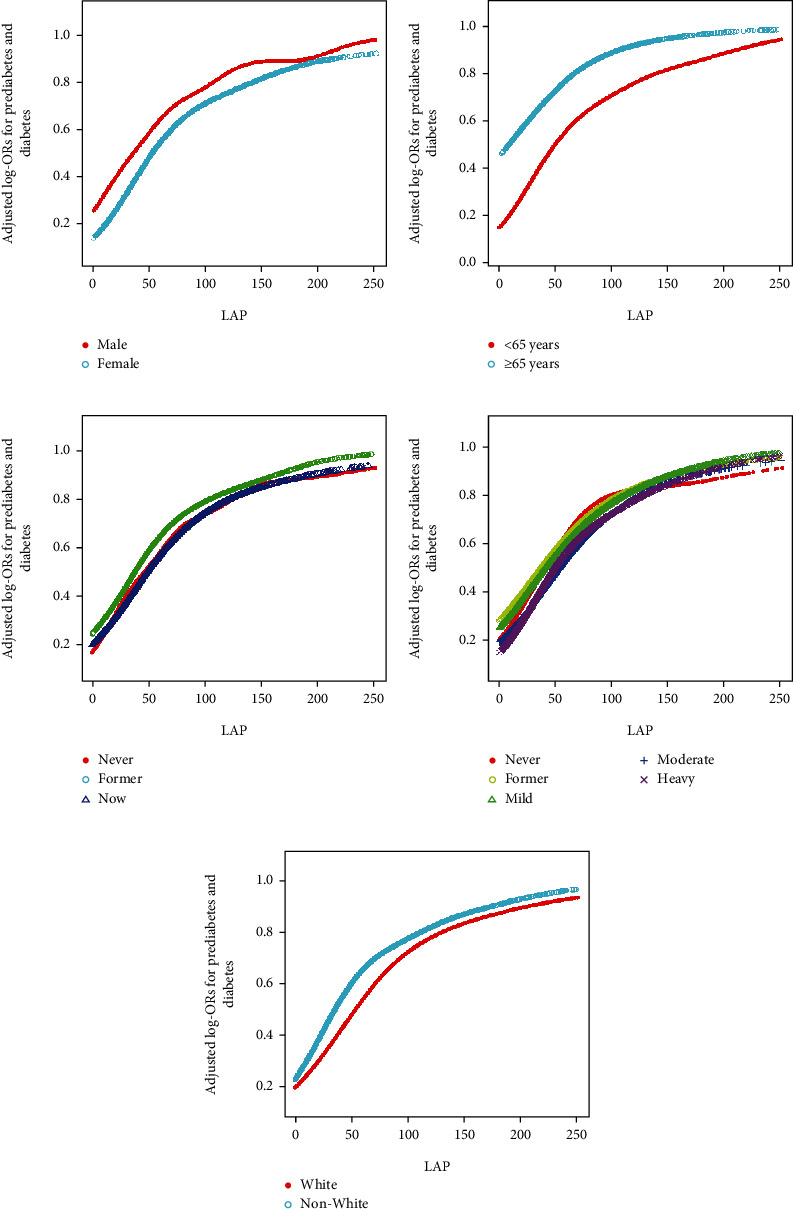
Stratified analyses (by (a) sex, (b) age, (c) smoking, (d) drinking, and (e) race/ethnicity) between LAP and the prevalence of prediabetes and diabetes using generalized additive model and smooth curve fittings. Note: Each generalized additive model and smooth curve fitting was adjusted for all factors, including age, sex, race/ethnicity, education level, smoking, drinking, poverty income ratio, METs/week, SBP, eGFR, TG, TC, lipid-lowering medications, and antihypertensive medications, except for the stratification factor itself. Abbreviations: LAP, lipid accumulation product; MET, metabolic equivalent of task; SBP, systolic blood pressure; eGFR, estimated glomerular filtration rate; TG, triglyceride; TC, total cholesterol.

As part of the sensitivity analysis, the relationship between LAP and the onset of prediabetes and diabetes was further explored through a hierarchical analysis using smoothed curve fitting. This analysis demonstrated a positive association at each layer of variable stratification. Additionally, the nonlinear relationship between LAP and the risk of prediabetes and diabetes, which was initially observed in Figure 2 and detailed in Table 3, was also confirmed in each stratum as shown in Figure 4.

## 4. Discussion

Our research, based on the NHANES database (1999–2018), has uncovered noteworthy insights into the link between LAP and the likelihood of prediabetes and diabetes. We noted a direct association between LAP and markers indicative of a risk for prediabetes and diabetes, persisting even with adjustments for confounders. Moreover, we detected a nonlinear connection with a turning point at 68.1 in the relationship between LAP and these conditions. These findings imply that LAP might be effectively used as an indicator for monitoring prediabetes and diabetes.

The association between the components of the LAP—WC and TG levels—and the risk of diabetes is increasingly evident in contemporary research. Kahn pioneered the LAP concept, drawing from frequency distributions of adult WC and TG levels [4]. He posited that LAP might surpass BMI in accurately signaling lipid accumulation and diabetes susceptibility [20]. Echoing this, Ayundini et al., through a PRISMA-guided systematic review, scrutinized the LAP–diabetes interplay, spotlighting LAP's potential as a diabetes predictor [21]. Khanmohammadi et al.'s work, a systematic review and meta-analysis, compared LAP with other anthropometric measures in assessing risks for hypertension, diabetes, and mortality. Their findings endorsed LAP as a cost-effective, superior tool over traditional measures like BMI and WC [22]. Wakabayashi and Daimon, studying Japanese workers aged 35–40, discovered a significant link between LAP and the risk of diabetes and hypertension across both genders [23]. Yan et al. investigated how LAP trajectories over 5 years relate to diabetes onset in Chinese adults, finding that elevated LAP trajectories indicate a higher diabetes risk, irrespective of initial LAP levels [24]. In a similar vein, Tian et al. compared LAP and BMI in a Chinese adult cohort, determining that LAP is a superior predictor of diabetes compared to BMI [25].

LAP, as a composite index, effectively encapsulates the metabolic disturbances characteristic of insulin resistance and obesity, both of which are critical precursors in the development of diabetes [26]. WC, as a measure of central adiposity, is directly correlated with insulin resistance, a key factor in disturbed glucose homeostasis [27–29]. Elevated TG levels, another component of LAP, reflect altered lipid metabolism, which exacerbates insulin resistance and disrupts normal glucose metabolism [30]. This dual representation within LAP allows for a more nuanced and comprehensive assessment of diabetes risk compared to traditional measures, making it a valuable tool in both clinical and epidemiological settings for the identification and management of individuals at heightened risk of developing diabetes. However, the focus on prediabetes, a crucial stage in diabetes development, has been relatively recent. Prediabetes, a symptomless, moderately hyperglycemic condition, can escalate to diabetes if undiagnosed [31]. Our study hence amalgamated prediabetes and diabetes as outcomes to probe their LAP linkage. We discovered a marked 5.14 times elevation in prediabetes and diabetes risk in the highest LAP group relative to the lowest. In summary, our analysis solidified a consistent positive correlation between LAP and both prediabetes and diabetes.

Our analysis revealed a possible nonlinear association between LAP and the risk of prediabetes and diabetes. Yang et al.'s research uncovered a dose–response correlation between the LAP index and the onset of diabetes, indicating that in the nonobese Korean population, a higher LAP index quartile correlates with an increased diabetes risk [32]. Nonetheless, the study's approach to assessing nonlinear relationships was limited to comparing effect values between different bisector groups, without employing GAMs or restricted cubic splines, which are more specific for analyzing nonlinear relationships [33, 34]. Liu et al.'s retrospective study on a Japanese cohort also noted a similar nonlinear relationship between LAP and new-onset diabetes, with an increased risk observed particularly before the inflection point of 46.38 [8]. This finding diverges from our results. In their study, Liu et al. focused exclusively on diabetes mellitus as an outcome, not considering patients in the prediabetic stage who might later develop diabetes. Moreover, their diagnosis relied on FPG levels (≥ 7 mmol/L), HbA1c percentages (≥ 6.5%), or self-reported data, excluding the OGTT, potentially leading to an underestimation of diabetes prevalence. The study's limitation to a Japanese demographic also raises questions about the applicability of its findings to other ethnic groups, which may differ in terms of race, age, and lifestyle. Similarly, Deng et al.'s study in a Chinese hypertensive population found a significant nonlinear relationship between LAP and diabetes [35]. Similarly, Xu et al., in a study involving 15,717 participants from rural China aged 35 and above, used restricted cubic spline models and discovered a potential nonlinear relationship between LAP and diabetes risk [36]. However, both Deng and Xu used only restricted cubic spline analysis and did not conduct parametric two-part logistic regression to identify precise inflection points, limiting the potential clinical significance of the nonlinear relationship [35, 36]. Collectively, these studies suggest the existence of a demographic experiencing a more rapid increase in diabetes risk before a certain inflection point, aligning with our study's conclusions. Additionally, TG, a component of LAP, and other similar biomarkers have nonlinear relationships with diabetes or prediabetes, further supporting our findings [12, 13, 37–39]. Thus, verifying this nonlinear relationship in the US population, considering both prediabetes and diabetes as outcomes, is crucial.

Our study found that after the inflection point of 68.1, the rate of increase in the risk of prediabetes and diabetes decreases, challenging conventional beliefs and indicating a complex interplay between lipid accumulation and glucose metabolism. This could be explained by various physiological mechanisms. As LAP rises to this critical point, the body's metabolic systems, especially those governing insulin sensitivity and glucose metabolism, might near a saturation limit. Beneath this point, the body could be more reactive to lipid accumulation changes, significantly influencing insulin resistance and glucose metabolism [40, 41]. Furthermore, the body employs mechanisms to stabilize glucose levels. These may operate more effectively under the threshold. However, surpassing the inflection point could overload these compensatory responses, resulting in a diminished rate of risk increase [42].

Moreover, combining the inflection point at 68.1 with the baseline characteristic differences observed in Table 1, we can see that individuals above this inflection point are primarily concentrated in the Q4 group. Specifically, people with LAP values above 68.1 tend to be older and have a higher proportion of non-Hispanic whites, lower education levels, higher smoking rates, worse economic status, higher blood pressure and lipid levels, and poorer kidney function. On the one hand, this could be due to immunosenescence in the elderly, where the innate immune system is enhanced and adaptive immunity is weakened, potentially affecting their response to inflammation and diabetes [43]. Additionally, different genetic backgrounds and lifestyle habits among various races can influence the development speed of diabetes, as confirmed by previous studies [44]. Smoking can cause various bodily damages that might mask some symptoms of diabetes, thus hiding its progression [45]. Poor economic status may prevent individuals from affording healthcare costs, possibly leading to a slower rate of risk increase for prediabetes and diabetes [46]. Medications for hypertension and high cholesterol may also impact blood glucose levels, affecting the risk of prediabetes and diabetes [47]. Finally, poor kidney function can impact metabolic regulation, potentially affecting glucose metabolism [48]. Overall, considering the characteristics of both groups, clinical and public health strategies should tailor interventions to the specific needs of different populations.

The stratified analysis undertaken to assess the effect of LAP independently from the covariates previously mentioned yielded notable findings. The results derived from both the forest plot–based logistic regression and the subgroup analysis of the GAMs consistently indicated a robust positive association between LAP levels and the risk of both prediabetes and diabetes. This association was observed irrespective of variations in sex, age, smoking, drinking, and race/ethnicity. The consistency of these results across different subgroups highlights the reliability and generalizability of the findings. It suggests that the relationship between LAP and the increased risk of prediabetes and diabetes is not significantly influenced by these demographic and lifestyle factors. This implies that LAP could be a universally relevant marker for predicting the risk of these conditions, making it a valuable tool for screening and preventative strategies in diverse populations.

These findings have important potential clinical implications. Firstly, LAP, as a convenient and cost-effective screening tool, can be used to detect individuals at high risk for prediabetes and diabetes early, thereby facilitating the formulation of early intervention and prevention strategies. By measuring WC and fasting TG levels during routine checkups, healthcare providers can quickly calculate LAP values and identify those at high risk. This screening method not only can improve the early diagnosis rate of the disease but also can reduce the number of undiagnosed diabetes cases, thereby reducing the incidence of diabetes-related complications. Additionally, considering the nonlinear relationship between LAP and prediabetes and diabetes, LAP can be used to optimize the allocation of medical resources, focusing on personalized prevention and management measures for different types of populations, thereby improving overall public health outcomes. Therefore, we recommend the widespread adoption of LAP in clinical practice as a screening tool to enhance the early detection and intervention for diabetes and prediabetes.

However, caution is advised in interpreting the findings due to several limitations. As a cross-sectional observational study, it does not establish causality or directionality. Despite thorough adjustments for confounding variables, the possibility of other influencing factors cannot be entirely eliminated. Moreover, the nonlinear correlation between LAP and prediabetes and diabetes is still a debated issue in numerous studies. Therefore, future longitudinal studies are recommended to provide more robust evidence for the relationship between LAP, prediabetes, and diabetes.

## 5. Conclusion

In conclusion, our study contributes to the growing body of evidence supporting the use of LAP as a reliable marker for predicting diabetes and prediabetes. It underscores the need for early detection and targeted interventions, particularly for individuals at high risk. Future research should focus on longitudinal studies to better understand the causal mechanisms and to explore the potential of LAP in guiding personalized prevention and treatment strategies for metabolic diseases.

## Figures and Tables

**Table 1:** Baseline characteristics of subjects.

**Characteristics**	**LAP**				**p** ** value**
	**Q1 (0.21–22.34)** **n** = 6030	**Q2 (22.34–41.51)** **n** = 6030	**Q3 (41.51–72.63)** **n** = 6030	**Q4 (72.63–251.02)** **n** = 6031	
Age (years)	37.83 ± 18.28	48.47 ± 19.03	51.43 ± 18.32	51.78 ± 17.40	< 0.001
Sex (%)					0.307
Male	49.29	47.79	48.94	48.07	
Female	50.71	52.21	51.06	51.93	
Race/ethnicity (%)					< 0.001
Non-Hispanic White	37.78	42.85	42.85	50.06	
Non-Hispanic Black	27.38	22.49	19.25	12.82	
Mexican American	14.64	16.73	20.96	22.83	
Others	20.20	17.93	16.93	14.29	
Educational level (%)					< 0.001
Less than high school	24.00	26.56	30.03	31.75	
High school	24.34	22.62	23.08	24.93	
More than high school	51.66	50.82	46.88	43.31	
Smoking (%)					< 0.001
Never	60.74	56.41	53.05	48.95	
Former	17.30	23.53	27.36	30.74	
Now	21.97	20.06	19.59	20.31	
Drinking (%)					< 0.001
Never	14.94	14.11	14.22	15.35	
Former	10.65	16.30	19.47	22.18	
Mild	35.56	34.84	32.60	30.79	
Moderate	18.13	15.30	13.42	12.28	
Heavy	20.72	19.45	20.29	19.40	
Poverty income ratio (%)					< 0.001
Low	31.59	29.70	30.38	33.72	
Medium	37.10	37.50	40.12	38.44	
High	31.30	32.79	29.51	27.84	
METs/week (%)					0.544
Low	95.13	94.91	95.30	95.31	
Moderate	2.55	2.95	2.76	2.88	
Vigorous	2.32	2.14	1.94	1.81	
SBP (mmHg)	116.09 ± 16.63	122.67 ± 19.09	125.52 ± 19.15	127.16 ± 18.93	< 0.001
DBP (mmHg)	66.87 ± 10.89	68.93 ± 12.05	70.36 ± 12.02	71.34 ± 12.86	< 0.001
eGFR (mL/min/1.73 m^2^)	106.15 ± 22.42	96.17 ± 23.88	93.15 ± 24.50	92.15 ± 25.41	< 0.001
TG (mg/dL)	60.00 (47.00–76.00)	87.00 (71.00–107.00)	121.50 (100.00–149.00)	195.00 (154.00–251.00)	< 0.001
TC (mg/dL)	174.18 ± 35.99	189.93 ± 38.67	198.27 ± 40.52	208.57 ± 43.79	< 0.001
Lipid-lowering medications (%)	6.54	15.61	20.39	23.83	< 0.001
Antihypertensive medications (%)	11.88	25.32	33.89	41.89	< 0.001
Glucose metabolism state (%)					< 0.001
No prediabetes	70.33	51.36	38.57	29.43	
Prediabetes	24.54	36.30	40.55	39.89	
Diabetes	5.12	12.34	20.88	30.67	

Abbreviations: DBP, diastolic blood pressure; eGFR, estimated glomerular filtration rate; LAP, lipid accumulation product; MET, metabolic equivalent of task; SBP, systolic blood pressure; TC, total cholesterol; TG, triglyceride.

**Table 2:** Relationship between LAP and prediabetes and diabetes in different models.

**LAP**	**Model 1**	**Model 2**	**Model 3**
LAP/10	1.16 (1.15, 1.16)	1.13 (1.12, 1.14)	1.22 (1.20, 1.25)
LAP (quartile)			
Q1 (0.21–22.34)	Reference	Reference	Reference
Q2 (22.34–41.51)	2.25 (2.08, 2.42)	1.60 (1.48, 1.74)	1.64 (1.46, 1.83)
Q3 (41.51–72.63)	3.78 (3.50, 4.07)	2.55 (2.34, 2.77)	2.69 (2.38, 3.04)
Q4 (72.63–251.02)	5.68 (5.26, 6.15)	4.22 (3.87, 4.60)	5.14 (4.34, 6.07)
*p* for trend	< 0.0001	< 0.0001	< 0.0001

*Note:* Model 1: nonadjusted. Model 2: adjusted for age, sex, race/ethnicity, and education level. Model 3: adjusted for all variables in Model 2 and smoking, drinking, poverty income ratio, METs/week, SBP, eGFR, TG, TC, lipid-lowering medications, and antihypertensive medications.

Abbreviations: eGFR, estimated glomerular filtration rate; LAP, lipid accumulation product; MET, metabolic equivalent of task; SBP, systolic blood pressure; TC, total cholesterol; TG, triglyceride.

**Table 3:** Threshold effect analysis of LAP on prediabetes and diabetes using a two-part logistic regression model.

**LAP/10**	**Adjusted OR** ^a^ ** (95% CI)**	**p** ** value**
Model I		
Fitting by the standard linear model	1.21 (1.19, 1.23)	< 0.001
Model II		
Inflection point	68.1	
< 68.1	1.32 (1.29, 1.35)	< 0.001
> 68.1	1.14 (1.11, 1.16)	< 0.001
Log-likelihood ratio	/	< 0.001

Abbreviations: eGFR, estimated glomerular filtration rate; LAP, lipid accumulation product; MET, metabolic equivalent of task; SBP, systolic blood pressure; TC, total cholesterol; TG, triglyceride.

^a^Adjusted for age, sex, race/ethnicity, education level, smoking, drinking, poverty income ratio, METs/week, SBP, eGFR, TG, TC, lipid-lowering medications, and antihypertensive medications.

## Data Availability

Publicly available datasets were analyzed in this study. This data can be found on the NHANES website (https://www.cdc.gov/nchs/nhanes/index.htm).

## Supporting Information

Supporting Information Additional supporting information can be found online in the Supporting Information section. The supporting information provides detailed descriptions and methods related to the calculation of covariates used in this study. These materials include the classification and calculation methods for smoking status, alcohol use, physical activity, and PIR. These supporting tables provide comprehensive information necessary to replicate the analyses conducted in this study.
