# Supplementary material for: Lipid Accumulation Product as a Predictor of Prediabetes and Diabetes: Insights From NHANES Data (1999–2018)
Source: J Diabetes Res. 2024 Nov 11;2024:2874122. doi: 10.1155/2024/2874122 (PMC11573446; doi:10.1155/2024/2874122)
Supplement: Supporting Information — Additional supporting information can be found online in the Supporting Information section. The supporting information provides detailed descriptions and methods related to the calculation of covariates used in this study. These materials include the classification and calculation methods for smoking status, alcohol use, physical activity, and PIR. These supporting tables provide comprehensive information necessary to replicate the analyses conducted in this study. [file 2874122.f1.docx]

**Table S1. Classification of Smoking Status in NHANES**

| Smoking Status | Definition | Questionnaire Items | Question | Response Options |
| --- | --- | --- | --- | --- |
| Never Smokers | Never smoked or smoked fewer than 100 cigarettes in their lifetime | SMQ020 | Have you smoked at least 100 cigarettes in your entire life? | No |
| Former Smokers | Smoked at least 100 cigarettes in their lifetime but do not currently smoke | SMQ020, SMQ040 | Have you smoked at least 100 cigarettes in your entire life? Do you now smoke cigarettes? | SMQ020: Yes SMQ040: Not at all |
| Current Smokers | Currently smoke every day or some days | SMQ020, SMQ040 | Have you smoked at least 100 cigarettes in your entire life? Do you now smoke cigarettes? | SMQ020: Yes SMQ040: Every day or Some days |

**Notes:** Detailed Explanation of Questionnaire Items

- SMQ020: Have you smoked at least 100 cigarettes in your entire life?
- Response Options:
- Yes
- No
- Refused
- Don't know
- SMQ040: Do you now smoke cigarettes?
- Response Options:
- Every day
- Some days
- Not at all
- Refused
- Don't know

**Table S2. Classification of Alcohol Use in NHANES**

| Drinking Level | Definition | Questionnaire Items | Response Options and Calculation |
| --- | --- | --- | --- |
| Heavy Alcohol User | - ≥3 drinks per day for females  - ≥4 drinks per day for males  - Binge drinking on 5 or more days per month | ALQ130 ALQ141Q ALQ270 | - ALQ130: ≥3 drinks/day (females) or ≥4 drinks/day (males)  - ALQ141Q or ALQ270: Binge drinking ≥60 days/year |
| Moderate Alcohol User | - ≥2 drinks per day for females  - ≥3 drinks per day for males  - Binge drinking on ≥2 days per month | ALQ130 ALQ141Q ALQ270 | - ALQ130: ≥2 drinks/day (females) or ≥3 drinks/day (males)  - ALQ141Q or ALQ270: Binge drinking ≥24 days/year |
| Mild Alcohol User | - Does not meet the above criteria | ALQ130 ALQ141Q ALQ270 | - ALQ130: <2 drinks/day (females) and <3 drinks/day (males)  - ALQ141Q or ALQ270: Binge drinking <24 days/year |

**Notes:** Detailed Explanation of Key Items

- ALQ130: Average Number of Drinks per Day in the Past 12 Months
- Question: "During the past 12 months, on those days that you drank alcoholic beverages, on average, how many drinks did you have?"
- Response Options: Enter the number of drinks. (If less than one drink, enter '1').
- ALQ141Q: Frequency of Binge Drinking in the Past 12 Months
- Question: "In the past 12 months, on how many days did you have 4/5 or more drinks of any alcoholic beverage?"
- Response Options: Enter the number of days. (For males, 5 or more drinks; for females, 4 or more drinks).
- ALQ270: Frequency of Heavy Drinking in a Short Period in the Past 12 Months
- Question: "During the past 12 months, about how often did you have 4/5 or more drinks in a period of two hours or less?"
- Response Options: Enter the number of days. (For males, 5 or more drinks; for females, 4 or more drinks).

**Table S3. NHANES Physical Activity Calculation Using METs**

| Questionnaire Items | Description | MET Value | Calculation |
| --- | --- | --- | --- |
| PAQ610 | Days/week vigorous-intensity work-related physical activity | 8.0 | (PAQ610 * PAD615) / 60 |
| PAD615 | Minutes/day vigorous-intensity work-related physical activity | - | Included in calculation for PAQ610 |
| PAQ625 | Days/week moderate-intensity work-related physical activity | 4.0 | (PAQ625 * PAD630) / 60 |
| PAD630 | Minutes/day moderate-intensity work-related physical activity | - | Included in calculation for PAQ625 |
| PAQ655 | Days/week vigorous-intensity recreational physical activity | 8.0 | (PAQ655 * PAD660) / 60 |
| PAD660 | Minutes/day vigorous-intensity recreational physical activity | - | Included in calculation for PAQ655 |
| PAQ670 | Days/week moderate-intensity recreational physical activity | 4.0 | (PAQ670 * PAD675) / 60 |
| PAD675 | Minutes/day moderate-intensity recreational physical activity | - | Included in calculation for PAQ670 |

**Notes:** Calculation Formula

- METs/week = Weekly Activity Minutes × MET value

**Table S4. Details for PIR Calculation**

| Description | Questionnaire Items | Years | Question | Details |
| --- | --- | --- | --- | --- |
| Annual Family Income | INDFMINC | 1999-2006 | "What is your total combined family income in the past 12 months?" | Captures the total income of all family members over the past year. |
| Annual Family Income | INDFMIN2 | 2007-2018 | "What is your total combined family income in the past 12 months?" | Same as INDFMINC but used for later years. |
| Total Annual Household Income | INDHHIN2 | 1999-2018 | "What is the total income for all household members in the past year?" | Represents the total annual income of all individuals in the household. |
| Ratio of Family Income to Poverty | INDFMPIR | 1999-2018 | Calculated based on income and poverty threshold | The ratio of family income to the poverty threshold specific to the survey year. |
| Total Number of People in the Household | DMDHHSIZ | 1999-2018 | "How many people are living in your household?" | Includes all people living in the household. |
| Total Number of People in the Family | DMDFMSIZ | 1999-2018 | "How many people are living in your family?" | Includes all family members related by birth, marriage, or adoption living together. |

**Notes:** Calculation of PIR

- PIR = Family Income / Poverty Threshold
